# Haptens Optimization Using Molecular Modeling and Paper-Based Immunosensor for On-Site Detection of Carbendazim in Vegetable Products

**DOI:** 10.3390/bios15090625

**Published:** 2025-09-19

**Authors:** Wenjing Chen, Zhuzeyang Yuan, Kangliang Pan, Yu Wang, Xiaoqin Yu, Tian Guan, Jiahong Chen, Hongtao Lei

**Affiliations:** 1 Guangdong Provincial Key Laboratory of Food Quality and Safety/Nation-Local Joint Engineering Research Center for Machining and Safety of Livestock and Poultry Products, South China Agricultural University, Guangzhou 510642, China; wenjingchen2000@163.com (W.C.); gordon_william@163.com (Z.Y.); pkkliang@163.com (K.P.); guantian@scau.edu.cn (T.G.); 2 Guangdong Laboratory for Lingnan Modern Agriculture, Guangzhou 510642, China; 3 Guangzhou Institute of Food Inspection, Guangzhou 511410, China; xxwangyu@163.com; 4 Key Laboratory of Baijiu Supervising Technology for State Market Regulation, Sichuan Institute of Food Inspection, Chengdu 611730, China; yuxiaoqinhuan@163.com

**Keywords:** carbendazim, computer-aided hapten design, specific antibody, lateral flow immunochromatographic immunoassays

## Abstract

Carbendazim is a benzimidazole fungicide widely used in the prevention and control of vegetable diseases. However, if misused, it may result in residues in agricultural products, not only reducing vegetable quality but also posing potential risks to human health. Currently, the on-site rapid detection technology for carbendazim still faces challenges, including insufficient antibody specificity and low sensitivity, which hinder its ability to meet practical regulatory requirements. Therefore, this study screened a rational hapten structure by applying a computer-aided hapten design and obtained a specific antibody. Compared to previous studies, the cross-reactivity rate of the antibody with thiabendazole-methyl was less than 0.1%, and the cross-reactivity rate with 2-aminobenzimidazole was 52.7% lower than that of the existing reported antibodies, which significantly improved the detection specificity of the method. Based on a high-specificity antibody, a gold nanoparticle-based lateral flow immunoassay (AuNPs-LFIA) for carbendazim was established. The detection limits of green beans and leeks are 3.80 μg/kg and 1.80 μg/kg, respectively, which still maintain high specificity in complex samples. Good agreement was also demonstrated between the results of blind samples detected by AuNPs-LFIA and LC-MS/MS, respectively. The establishment of AuNPs-LFIA provides an effective solution for the rapid and specific detection of carbendazim.

## 1. Introduction

Carbendazim (CBZ), a benzimidazole fungicide, is widely used around the world to control fungal diseases in vegetable products. However, its potential for leaving behind residues poses a serious threat to food safety and the ecosystem [1]. It is concerning that some unscrupulous producers, in their pursuit of disease prevention, not only overdose but also deliberately shorten the safety interval and illegally apply prohibited drugs to vegetable products [2]. It causes CBZ residues in agricultural products to exceed the standard frequently [3]. There is a large accumulation of CBZ in the human body, and its residue will cause neurotoxicity (dizziness, convulsions) and organ damage, as well as damage to soil microbiota and aquatic ecosystems [4,5]. Residues of CBZ in vegetable products must be strictly controlled below the safety limit standards [6]. According to the national standard of the People’s Republic of China (GB 2763-2021) [7], the Maximum Residue Limits (MRLs) of CBZ in vegetable products are 0.05 mg/kg–2 mg/kg. Therefore, it is necessary to develop efficient and reliable detection for CBZ residues.

The analytical techniques currently used for the detection of CBZ are mainly instrumental methods, including high-performance liquid chromatography (HPLC), liquid chromatography-tandem mass spectrometry (LC-MS/MS), as well as molecularly imprinted aptamer sensors and surface-enhanced Raman scattering sensor methods, with high accuracy and precision [8,9,10,11]. However, these methods often suffer from the disadvantages of expensive instruments, the need for skilled personnel, complex sample pre-treatment, and unsuitability for rapid screening in the field [12]. Immunoassay techniques have the advantages of high sensitivity, good stability, and ease of use [13]. In particular, lateral flow immunochromatography (LFIA) is ideal for large-scale rapid screening because of its significant advantages, such as cost-effectiveness and field applicability [14]. However, existing immunoassays (e.g., LFIAs) still face the problem of high antibody cross-reactivity in the detection of CBZ residues in vegetable products, which may lead to false-positive results [15]. To enhance the specificity of the assay, a computer-assisted chemical analysis technology was employed, including analysis of the conformation, chemical parameters, and electrostatic properties of haptens, which is helpful to reduce the cross-reactivity between the antibody and non-target substances by accurately optimizing the design of antigenic epitopes, thus ensuring the reliability of the assay results.

**Figure 1:**
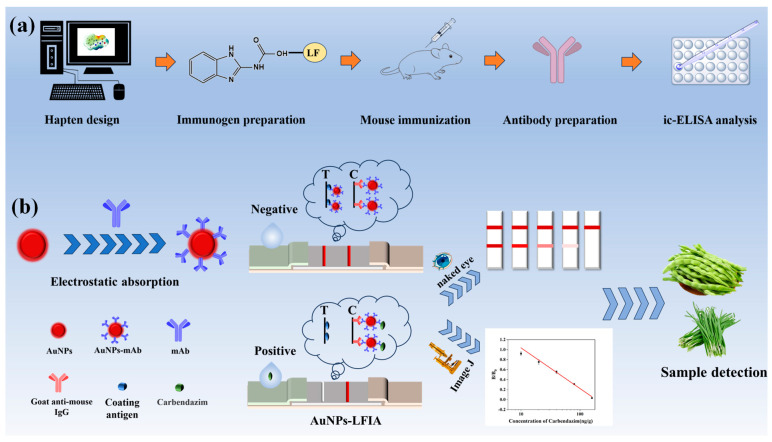
Schematic diagram of the principle of computerized chemistry-assisted analysis for monoclonal antibody preparation and AuNPs-LFIA. (**a**) Molecular simulation analysis and antibody preparation. (**b**) Schematic diagram of the AuNPs-LFIA.

Currently, the preparation of specific antibodies mainly relies on traditional antigen design methods based on experience [16]. However, this method has limitations, such as blind antigenic epitope design, which may lead to high antibody cross-reactivity rates. In particular, the problem of interference with structural analogs has not yet been effectively resolved [17]. This study introduced computer-assisted antigen design to the traditional antibody preparation system. Key factors, such as superposition of the lowest-energy conformations, chemical structure parameters, charge distribution, and surface electrostatic potential, were analyzed to select optimal haptens and use them to prepare immunogens for immunizing mice to produce highly specific antibodies (Figure 1a). Based on the prepared monoclonal antibody, a gold nanoparticle-based lateral flow immunoassay (AuNPs-LFIA) with high specificity and sensitivity was established and applied to vegetable products (Figure 1b).

## 2. Materials and Methods

### 2.1. Reagents and Instruments

CBZ (≥98%), N, N-dimethylformamide (DMF), N, N′-carbonyldiimidazole (CDI), mono-methyl terephthalate, 2-aminobenzimidazole, and succinic acid were received from BiDe Pharma Tech Co., Ltd. (Shanghai, China). Bovine serum albumin (BSA), N-(3(dimethylamino) propyl)-N′-ethylcarbodiimide (EDC), N-hydroxysuccinimide (NHS), and lactoferrin (LF) were procured from Sigma-Aldrich (St. Louis, MO, USA). The Unisart CN95 nitrocellulose membrane material was acquired from Sartorius Stedim Biotech (Germany). For lateral flow assembly, glass fiber sample pads (Catalog SB08), cellulose-based absorption matrices (CH37K), and vinyl polymer backing substrates (SMA31-40) were obtained from Liangxin Biotechnology (Shanghai, China). All precursor chemicals, including chloroauric acid trihydrate, sodium citrate tribasic, and poly(N-vinylpyrrolidone) (PVP), were obtained from Sinopharm Chemical (Shanghai, China). 250 kDa Plus Prestained Protein Marker (250 kDa) was purchased from Nanjing Vazyme Biotech Co., Ltd. (Nanjing, China).

The nitrocellulose (NC) filter membranes were sprayed by an XYZ 3060 Di. Both test and control lines were patterned onto nitrocellulose membranes using the Platform (BioDot, Irvine, CA, USA). Protein-hapten conjugation was verified using a NanoDrop 2000C ultraviolet spectrophotometer (Thermo Scientific, Yorba Linda, CA, USA). Gold nanoparticle morphology was characterized by transmission electron microscopy (Talos L120C, Thermo Scientific, Yorba Linda, CA, USA). Zeta potential was determined using a Malvern Zetasizer Nano ZS90, Malvern, UK). LC-MS/MS analyses were conducted on a SCIEX QTRAP 6500 system (Framingham, MA, USA) operating in MRM mode.

BALB/c female mice (6 weeks old) were purchased from Zhuhai Biotest Biotechnology Co., Ltd. (Zhuhai, China). All relevant animal experiments were conducted in accordance with the basic requirements of animal ethical review (Application number: 2024B094). Supporting materials can be found in Figure S1.

### 2.2. Computer-Aided Chemical Analysis

Computational chemistry was used to analyze the structural activity of the haptens. The two-dimensional structures of hapten 1–3 were first constructed using ChemDraw 18.0 (Winter St Waltham, MA, USA), transformed into three-dimensional conformations by Chem 3D, and optimized by the MM2 force field to obtain the lowest energy conformations. The optimized hapten 1–3 were molecularly overlaid with CBZ (target molecule) in Discovery Studio 2019. The PDB files were then processed using GaussView 5.0.8, converted to mol2 format, and then geometrically optimized based on the B3LYP/def-TZVP method. The molecular electrostatic potential was plotted by VMD in combination with Multiwfn, while the physical and chemical parameters, such as hydrophobic constants (LogP) and molecular weights, were obtained by ChemDraw calculations.

### 2.3. Preparation of Haptens and Artificial Antigens

**Figure 2:**
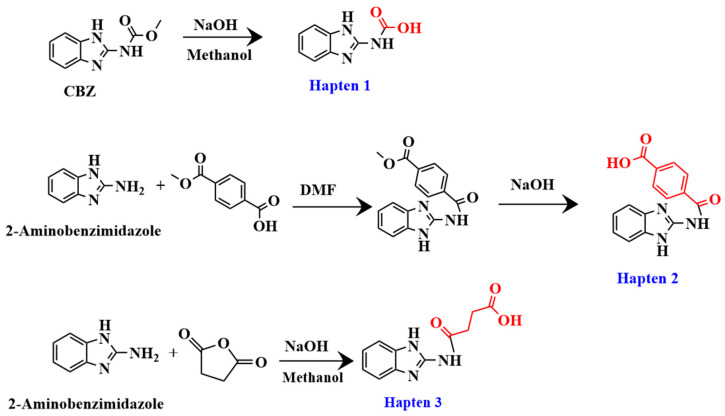
Synthesis route of haptens 1–3.

The synthesis processes of the haptens were shown in Figure 2, and specific methods for synthesizing haptens were shown in the supporting materials.

The hapten 1–3 were coupled to bovine serum albumin (BSA) or lactoferrin (LF) using the active ester method, respectively [18]. After purification, these artificial antigens were identified and concentration determined by Ultraviolet-visible (UV-Vis) spectroscopy scanning. The artificial antigens were stored in a refrigerator at −20 °C [19]. Hapten 1-LF, hapten 2-LF, and hapten 3-LF were used as immunogens, and hapten 1-BSA, hapten 2-BSA, and hapten 3-BSA were used as coating antigens.

### 2.4. Preparation of CBZ Monoclonal Antibodies

Each immunogen was used to immunize female BALB/c mice as described previously. For the first immunization, the immunogen was administered at a dosage of 100 μg emulsified in complete Freund’s adjuvant. Incomplete Freund’s adjuvant was used for the subsequent three-week booster immunizations with 50 μg of immunogen. One week after the final immunization, blood was taken from the tail of mice for sensitivity and specificity detection using the indirect competitive enzyme-linked immunosorbent assay (ic-ELISA). The mouse with the highest titer and inhibition rate toward CBZ was chosen for cell fusion to generate a monoclonal antibody (mAb).

The procedures of cell fusion, screening, and cloning were carried out according to previous literature [20], as per the protocol described in the supporting materials. After several subclones, the clones that achieved our goals were selected to produce ascites on a large scale. The collected ascites were purified by affinity chromatography columns and then confirmed by sodium dodecyl sulfate polyacrylamide gel electrophoresis (SDS-PAGE), and the performance of this mAb was assessed by ic-ELISA [21].

### 2.5. Preparation of AuNPs Immunoprobes

Citrate-capped gold nanocolloids were prepared by thermal reduction in auric chloride using sodium citrate as the reducing agent [22]. 100 mL of boiling ultrapure water was mixed with 4.0 mL of HAuCl_4_ solution (10 mg/mL) while being stirred. 8.0 mL of sodium citrate (10 mg/mL) was injected at boiling and reacted until the solution was burgundy in color. The AuNP suspension was equilibrated to 25 °C, volumetrically adjusted to 100 mL, and stored at 4 °C.

Gold nanoparticle immunoprobes are prepared using the electrostatic adsorption technique [23]. First, 12.5 μg of CBZ antibody was added to 1 mL of AuNP suspension in a centrifuge tube and reacted for 30 min at room temperature after the pH was adjusted with a 0.2 mol/L K_2_CO_3_ solution. Second, the mixture was then subjected to a 30 min blocking reaction with 50 μL of 10% BSA solution. Third, the solution was centrifuged at 9500× *g* for 15 min at 4 °C, and the supernatant was discarded. Nanoparticles were redispersed in 200 μL of optimized phosphate buffer (pH 7.4, 0.02 M) containing 0.5% BSA, 5% sucrose, and 0.5% Tween-20, with 0.03% proclin-300 added as preservative, and then stored at 4 °C. The coupling of antibodies and colloidal gold was determined by monitoring changes in zeta potential and combining the characteristics of UV-Vis spectroscopy absorption peaks.

### 2.6. Preparation of Test Strip

The lateral flow strip assembly incorporates four functional components: sample reception pad, NC film, polyvinyl chloride backing (PVC), and absorbent pad [24]. Coating antigen (T-line) and goat anti-mouse IgG (C-line) were applied to nitrocellulose membranes at a regulated rate of 0.8 μL/cm using a precision dispensing device (XYZ Biostrip). The produced NC film, sample pad, and absorbent pad were sequentially adhered to the PVC pad after being dried for 12 h at 37 °C. The test strips were then divided into parts that were 3.05 mm wide and kept as a backup at room temperature.

### 2.7. Sample Preparation

In this study, green beans and leeks were selected as samples for testing because they are the most widely used. After weighing 2 g of leek and green bean samples into a 10 mL centrifuge tube, 2 mL of 10% methanol was added. The mixture was vortexed for 1 min and then filtered through a 0.45 μm membrane filter. The resulting supernatant was collected and stored for subsequent analysis.

### 2.8. Test Procedure

First, 4 μL of AuNPs immunoprobes and 150 μL of sample extract were combined in microtiter wells and allowed to sit at room temperature for three minutes. Second, to allow for the chromatographic response, the test strip was placed vertically into the microtiter wells and left there for four minutes. Lastly, discard the sample pad and take the test strip out of the microtiter wells. As shown in Figure 1b, the outcomes were shown graphically. ImageJ 1.54p (Bethesda, MD, USA) was used to measure the test strips’ grayscale values. Then, the results were quantified by analyzing the color shades of the reactive areas on the strips.

The test results are as follows: when CBZ is not present in the sample, the probe is captured by the encapsulated antigen on the T-line, forming a visible red band. When CBZ is present in the sample, CBZ reacts competitively with the coating antigen, resulting in some or no CBZ-mAb being captured on the T-line. T-line. In other words, within the detection range, as the amount of CBZ in the sample is larger, the signal response is weaker [25].

### 2.9. Evaluation of Analytical Performance

#### 2.9.1. Sensitivity

A number of crucial factors were adjusted to optimize the AuNPs-LFIA’s sensitivity, including the coating antigen concentration, antibody dilution buffer, pH for labeling, and antibody amount for labeling. The cut-off value, calibration curve, and limits of detection (LOD) were all determined using triple spiking sample analysis conducted under ideal conditions to validate the AuNPs-LFIA sensitivity [26]. According to European Commission Resolution 2002/657/EC, LOD is defined as the mean plus three times the standard deviation (SD) of the background signal generated by 20 blank samples, and the cut-off value is the threshold response value set in the screening analysis to distinguish between potential positive and negative samples [27].

#### 2.9.2. Specificity

The specificity of the AuNPs-LFIA was detected by testing the cross-reactivity with other structural or functional analogs, including 2-aminobenzimidazole,2-ethyl-1H-benzimidazole, Thiabendazole, Benomyl, Thiophanate-Methyl, Diniconazole, Uniconazole, and Paclobutrazol. The cross-reactivity rate (CR) is the ratio of the half-inhibitory concentration (IC_50_) of the interference substance to be tested to the half-inhibitory concentration of the target analyte.

#### 2.9.3. Accuracy

The recovery tests by spiked sample and blind sample analysis were used to assess the AuNPs-LFIA’s accuracy [28]. The AuNPs-LFIA and LC-MS/MS were used for the simultaneous detection of CBZ samples. Detailed operation and parameters of this LC-MS/MS method can be found in the supporting materials. In the recovery experiment, the concentration of the analyte measured by the established method was plotted on the horizontal axis, while the concentration measured by LC-MS/MS was plotted on the vertical axis. A fitting curve was obtained through linear regression analysis, and the correlation coefficient (R^2^) was used to evaluate the consistency between the two methods. Samples with low, medium, and high amounts of CBZ were used to test recoveries. The sample recovery rate is the percentage of the detected concentration and the added concentration of the target drug in the spiked sample. The concentrations of CBZ added to the leek and green bean samples were 50, 100, and 300 μg/kg and 15, 35, and 135 μg/kg, respectively. The assay was repeated three times for each spiked sample. The accuracy of this method is evaluated by calculating the recovery rate. The recovery rate is defined as the ratio of the actual measured value to the spiked value. The precision was evaluated by calculating the SD and coefficient of variation (CV).

#### 2.9.4. Stability

To guarantee the test strips’ dependability during use and storage, the stability of the test strips and immunoprobes was detected. Therefore, in this study, low-temperature stability tests and accelerated stability tests were conducted. Herein, samples from the same batch were placed in sealed bags containing desiccant and stored at 4 °C and 37 °C, respectively. Samples were taken on days 0, 7, 14, 21, and 28 of the storage period. The color development performance and inhibition rate of the test strips were examined to evaluate their stability under different temperature conditions.

## 3. Results and Discussion

### 3.1. Computer-Aided Chemical Analysis

Computer-aided hapten design strategy can help to precisely prepare highly specific antibodies by systematically analyzing the physicochemical properties of drugs and target site characteristics [29]. In this study, a multidimensional antigen evaluation system was built by integrating crucial characteristics such as minimal energy conformation analysis, chemical structure parameter evaluation, charge distribution computation, and surface electrostatic potential. Through the synergistic optimization of characteristics including conformational stability, structural match, and electrostatic complementarity, the best hapten molecules may be precisely screened. It offers a foundation for molecular design in the creation of highly specific antibodies.

#### 3.1.1. Lowest-Energy Conformational Analysis

**Figure 3:**
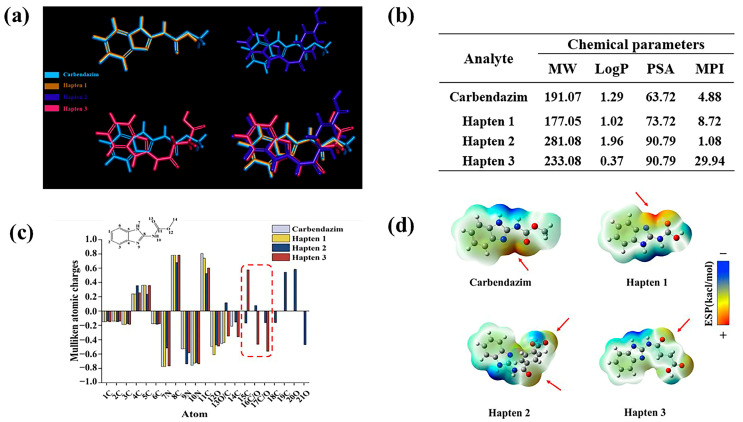
Analysis of hapten 1–3. (**a**) Lowest-Energy Conformational Analysis. (**b**) calculation of molecular weight (MW), hydrophobic parameters (Log P), polar surface area (PSA), and molecular polarity index (MPI) for phenacetin and haptens 1–3. (**c**) electrostatic interaction analysis. (**d**) molecular surface electrostatic potential analysis.

Highly specific antibody production is more likely to be induced by hapten structures that highly overlap with the lowest energy conformation of the target molecule [30]. CBZ was set as the target molecule for molecular stacking with haptens 1–3. The results are shown in Figure 3a, compared with hapten 1, hapten 3 adds a carboxyl group at C11, while hapten 2 adds a benzene ring moiety, which significantly reduces the rotatable bond flexibility. The addition of the moiety results in a substantial deflection of the overall conformation of hapten 2,3, resulting in a lower degree of overlap with the lowest energy conformation of CBZ. Therefore, the lowest energy conformation of hapten 1 has the highest overlap with CBZ.

#### 3.1.2. Chemical Structure Parameter Analysis

The oil-water partition coefficient is represented by the LogP value. Higher LogP values indicate that a molecule is more hydrophobic [31]. According to reports, moderate LogP maintains structural similarity to the target while improving the hapten’s presentation efficiency in immunized animals [32]. The chemical structure parameters were analyzed in Figure 3b. Thus, hapten 2 is the most hydrophobic, followed by hapten 1. It is noteworthy that the hydrophilicity of hapten 3 may potentially impact immunogenicity, as well as the recognition of hydrophobic regions by antibodies [33]. When binding to protein carriers, its greater polar surface area facilitates the formation of additional hydrogen bonds and electrostatic interactions [34,35]. This leads to improved immunogenicity and more stable binding to carrier proteins. With huge polar surface areas (PSA), haptens 2,3 have the greatest PSA, followed by hapten 1.

A molecule’s total polarity can be measured using the molecular polarity index (MPI); the higher the MPI value, the more polar the molecule is overall [36]. During the immunological response, the hapten’s contact with immune cells is enhanced when it is coupled with carrier proteins. This improves antibody specificity by allowing B cells to more effectively and precisely identify the epitope features of antigens [37]. In this work, the MPI of hapten 1 is 8.72, indicating that the molecule is moderately polar. Hapten 2 is the most hydrophobic molecule, as evidenced by its smallest MPI. When coupled with proteins, it has a tendency to aggregate, which hinders the hapten’s ability to distribute evenly or adhere firmly to the carrier protein’s surface [38]. Furthermore, on the one hand, its weak polarity tends to result in unstable binding to carrier proteins and decreased affinity for antibodies. On the other hand, it could result in nonspecific antibody binding to other hydrophobic substances during recognition and inadequate exposure of antigenic epitopes. Cross-reactions between these chemicals and other hydrophobic compounds are possible. Notably, hapten 3’s MPI is significantly higher than that of haptens 1,2, suggesting that its molecular hydrophobicity is excessive. This structure may lower specificity by preventing antibodies from correctly identifying the hapten’s structural characteristics [39]. Therefore, hapten 1 is likely to be the optimal hapten.

In conclusion, the formation of highly specific antibodies and the development of an immune response are facilitated by the high degree of similarity between the chemical structure parameters of hapten 1 and the target.

#### 3.1.3. Electrostatic Interaction Analysis

Mulliken atomic charge distribution is used to evaluate the charge characteristics of hapten epitopes. In this study, the electronic structure matching characteristics of the hapten and CBZ were analyzed. As shown in Figure 3c, the atomic charge distributions of CBZ and hapten 1 are similar. Haptens 2, 3 introduce different groups at C11, which results in significant differences in atomic charges at C15 and C/O16-17. Their electrostatic complementarity with the antibody binding pocket is directly impacted by this. Therefore, hapten 1 may be more advantageous in the production of highly specific antibodies.

#### 3.1.4. Molecular Surface Electrostatic Potential Analysis

As shown by the electrostatic potential map on the surface of the molecule (Figure 3d), both hapten 1 and CBZ have a large positive potential region at C11 (as indicated by the red arrow), which can form a strong electrostatic attraction with the antibody and enhance the affinity for the antibody [40]. A different electron-withdrawing group is added at C11 in haptens 2–3, which weakens the positive potential. Interestingly, hapten 2 unintentionally created a new positive potential region at the O20 location. This distinct charge distribution caused the antibody’s antigen-recognizing epitope to move from the benzimidazole ring to the benzene ring, which decreased the target’s specific recognition.

According to the findings of the aforementioned analysis, hapten 1 and CBZ are extremely comparable with regard to surface electrostatic potential, charge distribution, and lowest energy conformational stacking. The antibody generated in response to the immunization may have maximum specificity when hapten 1 is attached to a protein and utilized as an immunogen.

### 3.2. Characterization of Haptens and Artificial Antigens

For verification of successful synthesis of the haptens, HPLC-MS/MS and nuclear magnetic resonance (NMR) were used, and the results confirmed the successful synthesis of the three haptens, as shown in the Supporting Materials Section. The successful coupling of haptens 1–3 to carrier proteins was confirmed by UV-vis spectroscopy scanning [41]. When the carrier protein is combined with the hapten, the absorption peak of the artificial antigen is significantly shifted compared to the carrier protein. This shift is characterized by the absorption peak of the haptens (Figure S2).

### 3.3. Characterization of Antisera and mAb

As shown in Table S1, most mice exhibited a positive immune response, with titers ranging from 4000 to 32,000, indicating that the conjugates of haptens 1–3 used in the study were successfully prepared. The results showed that the antiserum potency and inhibition rate of hapten 1 were significantly higher than those of the other two haptens. Notably, every mouse that received the hapten1-LF vaccination developed particular antibodies, among which mouse No. 1 had the best antibody performance, and the antibody titer was 1:32,000. The rationale and viability of the suggested hapten design and screening method are confirmed by these results, which align with the predictions for haptens. Compared with the homologous coating antigen, the inhibition rate of the heterogeneous coating antigen combination on 1 g/mL CBZ drug was 78.64%, and the detection sensitivity was significantly improved. The monoclonal antibody was identified as an IgG1 subtype and was used in the development of LFIA after ascites was obtained by amplification culture and purified (Figure S3).

### 3.4. Characterization of AuNPs and AuNPs Immunoprobe

**Figure 4:**
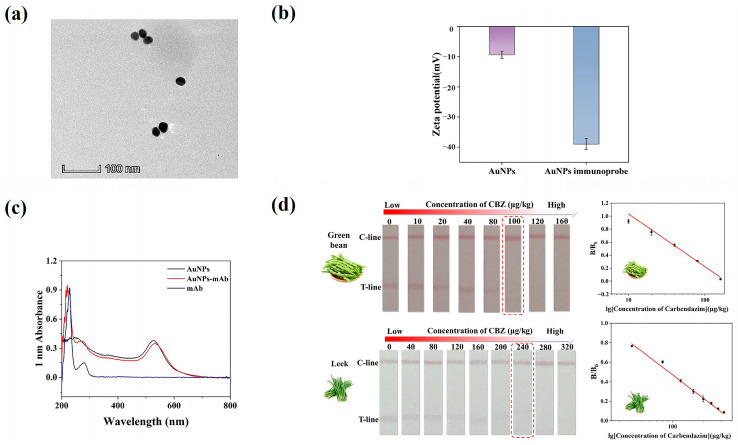
Characterization of AuNP and AuNP immunoprobes and Method sensitivity. (**a**) TEM image of AuNPs. (**b**) surface zeta potential of AuNPs and AuNPs immunoprobe. (**c**) The UV–vis spectroscopy of AuNPs and AuNPs immunoprobe. (**d**) Results of serial concentrations of CBZ in green beans and leeks, respectively; cutoff values are annotated with red dashed boxes.

The AuNPs were found to be regular, spherical particles with high dispersion and uniform size, according to the transmission electron microscope (TEM) study (Figure 4a). The average particle size was approximately 20 nm. When AuNPs and mAb are successfully coupled, the zeta potential usually changes significantly [42]. As shown in Figure 4b, the zeta potential decreased from −9.46 mV to −38.96 mV, indicating the successful synthesis of the AuNP immunoprobe. According to UV-Vis spectroscopy, after coupling with the antibody, the colloidal gold probe exhibits the characteristic peaks of both the antibody and the colloidal gold. Furthermore, a notable change in maximum absorbance suggests that the antibody and colloidal gold have effectively linked (Figure 4c).

### 3.5. Optimization of AuNPs-LFIA

#### 3.5.1. pH for Labeling

The stability of the immunoprobe and the effectiveness of antibody coupling are significantly impacted by the pH of the colloidal gold system [43]. Therefore, different quantities of 0.2 mol/L K_2_CO_3_ were added to the colloidal gold solution to change its pH. As shown in Figure S4a, when the pH was adjusted by adding 16 μL of 0.2 mol/L K_2_CO_3_, the test strips showed the best color rendering of red bands and the most significant inhibition effect, indicating that the binding efficiency of gold nanoparticles to the antibody was optimal under this pH condition.

#### 3.5.2. Antibody Amount for Labeling

In this study, the effect of antibody dose on the efficiency of AuNP-mAb coupling and detection performance (signal intensity and inhibition rate) was systematically investigated by gradient addition of mAb (5, 7.5, 10, 12.5, and 15 μg). The color intensity of the T line peaked, and the inhibition rate peaked at 12.5 μg of antibody. Ultimately, 12.5 μg of labeling antibody was selected (Figure S4b).

#### 3.5.3. Dilution Buffer of Antibody

Antibody dilution buffer guarantees the efficiency of colloidal gold-antibody coupling and improves test strip performance by optimizing conditions such as pH and ionic strength [44]. The performance of colloidal gold immunochromatographic strips was systematically assessed in this study about various antibody dilution buffers (0.01 mol/L borate buffer at pH 8.0, 0.01 mol/L phosphate buffer at pH 7.4, 0.5% bovine serum albumin (BSA) solution, and 0.01 mol/L Tris-HCl at pH 8.5). 0.01 mol/L Tris-HCl at pH 8.5 was found to be the ideal operating condition (Figure S4c).

#### 3.5.4. Concentration of Coating Antigen

In this work, we investigated the effects of varying encapsulated antigen concentrations on test strip performance. The antigen was diluted 10, 15, 20, 25, and 30 times from its initial concentration of 5.00 mg/mL. The findings demonstrated that excessive T-line binding to the gold standard antibody, caused by the high concentration of coating antigen (diluted 10 or 15 times) resulted in a weakening of the C-line coloration. At a 20-fold dilution, or 0.25 mg/mL of the coating antigen, the best color development intensity and inhibitory efficacy were achieved (Figure S4d). Therefore, this concentration was selected for further research.

### 3.6. Sensitivity

Leeks and green beans were chosen as study materials to confirm the accuracy of the well-established AuNPs-LFIA in identifying complicated matrices, such as those with high pigmentation or fiber content. Optimization of working conditions can be found in the supporting materials (Figures S5 and S6). We evaluated the sensitivity of the AuNPs-LFIA method for detecting CBZ in green beans and leeks under optimized conditions. The results showed that the coloration of the T-line gradually weakened as the concentration of CBZ increased. When the spiking amount in leeks and green beans reached 100 μg/kg and 240 μg/kg, respectively, the T-line vanished entirely (Figure 4d). This was first recognized as the cutoff point for a favorable or unfavorable interpretation. The LOD of the green beans sample was 3.80 μg/kg, and the linear range was 10.0–160.0 μg/kg. The LOD of the leek sample was 1.80 μg/kg, and the linear range was 40.0–320.0 μg/kg. Notably, the assay created in this study offers a more straightforward approach to ultrasensitive CBZ residue detection while meeting national sensitivity standards and facilitating quick, on-site detection with a streamlined process.

### 3.7. Specificity

**Figure 5:**
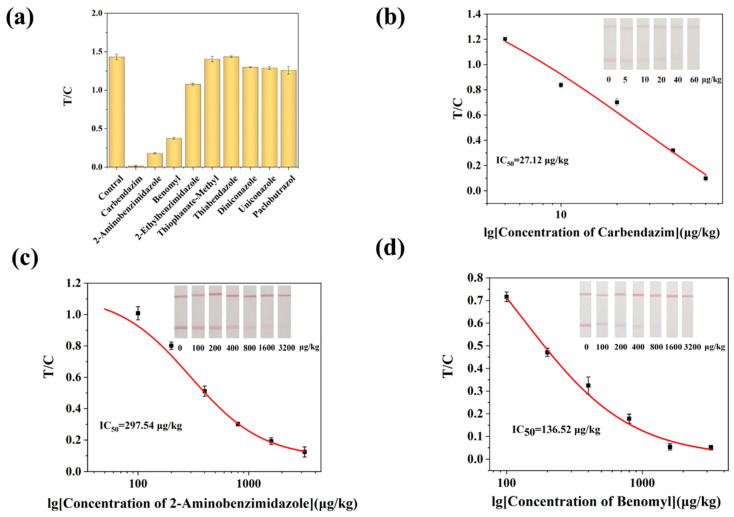
AuNPs-LFIA method specificity. (**a**) Selectivity evaluation of AuNPs-LFIA. (**b**) Calibration curve for the detection of CBZ by AuNPs-LFIA. (**c**) Calibration curves for the detection of CBZ (**b**), 2-aminobenzimidazole (**c**), and benomyl (**d**) by AuNPs-LFIA.

By comparing the cross-reactivity of AuNPs-LFIA and ic-ELISA with eight structural/functional analogs, the specificity of the procedure was confirmed in this investigation. As shown in Table 1, the antibody showed high specificity for CBZ using ic-ELISA. When the doping level was 1000 μg/kg, the T lines of the test strips for benomyl and 2-aminobenzimidazole in the AuNPs-LFIA method were not clearly defined. The T/C values of the remaining analogs were significantly higher than those of CBZ (Figure 5a). It demonstrates the respectable specificity of the proven AuNPs-LFIA. For these two medications, as well as CBZ, an AuNPs-LFIA standard curve was thus created. The results, as shown in Figure 5b–d, showed that the IC_50_ of 2-aminobenzimidazole, benomyl were 297.54 μg/kg and 136.52 μg/kg, respectively, in this method. This study used computer-aided design to create haptens, which produced antibody binding sites that beautifully match the benzimidazole compounds’ basic structure, especially the 2-methoxycarbonylamino group that connects the rings. Although benomyl possesses a benzimidazole core structure, its bulky side chain creates significant steric hindrance, reducing the likelihood of antibody recognition. Thiophanate-Methyl is not recognized by the antibody since it is a member of the substituted benzene fungicide class and does not have a benzimidazole component. In contrast to other research, the AuNPs-LFIA technique developed in this work not only dramatically lowers the cross-reactivity rate but also lowers the kinds of cross-reactants. This technique may be successfully used for the quick and precise identification of CBZ in food samples.

As shown in Table 2, this study compared the developed AuNPs-LFIA method with various CBZ detection methods reported in recent years, which cover different principles, in terms of key parameters, especially method specificity. The comparison shows that the prominent advantage of this method is not to pursue the lowest LOD, but to maintain good sensitivity while demonstrating significant specificity advantages. Compared to conventional techniques, the cross-reactivity rate of CBZ structural analogs has decreased, and the number of cross-reactive species has decreased, thanks to innovative immunogen design. Even with complicated samples, the approach can maintain high specificity detection because of the efficient reduction in matrix interference provided by the improved sample pretreatment strategy. Furthermore, the assay’s overall cost is greatly decreased by the immunogen synthesis strategy’s simplicity and effectiveness, as well as the small amount of organic solvents needed for sample extraction, making it ideal for field applications requiring quick screening.

### 3.8. Accuracy and Precision

The AuNPs-LFIA method developed in this study demonstrated excellent analytical performance with green beans and leeks. As shown in Figure S7, *R*^2^ > 0.99 indicates a strong linear correlation between the two techniques, demonstrating excellent consistency. In particular, the spiked recoveries for the leek samples ranged from 86.77% to 109.24% (with coefficients of variation between 2.74% and 8.04%) and for the green bean samples, from 90.82% to 117.73% (with coefficients of variation between 3.22% and 9.62%) (Table 3). The method’s good accuracy and intra-laboratory consistency were confirmed by the recoveries and coefficients of variation meeting the assay requirements. Thus, the established AuNPs-LFIA can rapidly detect CBZ residues in agricultural products.

### 3.9. Blind Sample Analysis

To verify the performance of the established method for practical applications, this study examined ten real samples of green beans and leeks using both AuNPs-LFIA and LC-MS/MS for parallel detection (Table S2). The results showed that the two assays produced highly consistent results. This comparison experiment confirmed that the AuNPs-LFIA method is accurate and practical.

### 3.10. Stability

As shown in Figure S8a, after 28 days of storage at 37 °C, there were differences in the color intensity of the test strips, with inhibition rates varying within 8%. As shown in Figure S8b, after 28 days of low-temperature storage, there were differences in the color intensity of the test strips, but the inhibition changes were not significant, with inhibition rates varying by less than 5%. It shows that the prepared AuNPs-LFIA system has excellent stability. The method ensures that the product has a stability of at least 0 to 28 days, which guarantees the reliability of the test strips in practical applications.

## 4. Conclusions

In conclusion, the antibody with high sensitivity and high specificity was rationally prepared by computer-assisted chemical analysis. Based on this, a straightforward AuNPs-LFIA was developed that can detect carbendazim quickly—within 10 min. Different from previous studies, the CR of the antibody with thiabendazole-methyl was less than 0.1%, and the CR with 2-aminobenzimidazole was 52.7% lower than that of the existing reported antibodies, which significantly improved the detection specificity of the method. The LODs of the method were 3.80 μg/kg and 1.80 μg/kg for the samples in green beans and leeks, with the recoveries of 90.82–117.73% and 86.77–109.24%, respectively. By integrating computational rational design with user-friendly paper-based diagnostic technology, this study provided a reliable and practical monitoring tool for detecting carbendazim residues. This strategy not only enhanced the accuracy and reliability of detection but also established a sustainable framework for developing next-generation rapid detection methods targeting other small-molecule pollutants, thereby supporting broader food safety and environmental protection efforts.

## Figures and Tables

**Table 1:** Cross-reactivities (CRs) of ic-ELISA.

Analytes	CAS	IC_50_ (ng/mL)	CR (%)
Carbendazim	10605-21-7	16.02	100
Benomyl	17804-35-2	189.96	8.43
Thiabendazole	148-79-8	>2000	<0.1
2-Aminobenzimidazole	149104-88-1	120.18	13.3
2-Ethylbenzimidazole	1848-84-6	>1500	<1
Thiophanate-Methyl	23564-05-8	>2000	<0.1
Diniconazole	836567-24-3	>2000	<0.1
Uniconazole	83657-22-1	>2000	<0.1
Paclobutrazol	76738-62-0	>2000	<0.1

**Table 2:** Comparison with other published detection methods for CBZ in food samples.

Method	Sample	LOD (μg/kg)	Testing Time (min)	Features	Reference
HPLC	Water	1.00	40	Using dispersive liquid–liquid microextraction	[8]
LC-MS/MS	Cabbage	0.5	>15	Complex pre-treatment and high cost	[9]
TRF-LFIA	Tobacco	0.56	-	CR (2-Aminobenzimidazole): 16.9% CR (Thiophanate-methyl): 25.9%	[12]
SERS-AuNPs	Oolong tea	100	>25	Complex pre-treatment and high cost	[45]
Electrochemical method	Apple Cabbage	11.74	-	On-site detection but low sensitivity	[46]
AuNPs-LFIA	Leek	0.18	<20	CR (2-Aminobenzimidazole): 19.32% CR (Thiophanate-methyl): 3.05%	[47]
Immunoassay Chip Detection	-	0.04	90	CR (Benomyl): 7.46% Long testing time	[48]
AIEFM@MPN-LFIA	Apple, pep-per, cucumber	0.019	-	significant cross-resistance with benomyl	[49]
AuNPs-LFIA	Green bean Leek	3.80 1.80	10	Easy operation, low cost, lower cross-reactivity, and fewer cross-reacting species.	This work

Note: “-” indicates no reference in the text.

**Table 3:** Analysis of samples of green beans and leeks artificially supplemented with carbendazim (*n* = 3).

Sample	Method	Spiked Levels (μg/kg)	Measured Level (μg/kg)	Recovery (%)	CV (%)
Green beans	AuNPs-LFIA	15	17.66 ± 1.70	117.73	9.62
		35	32.52 ± 1.36	92.91	4.18
		135	122.61 ± 3.95	90.82	3.22
	LC-MS/MS	15	15.23 ± 1.24	101.53	8.14
		35	36.39 ± 1.12	104.82	3.07
		135	128.41 ± 2.46	95.12	1.92
Leek	AuNPs-LFIA	50	54.62 ± 4.39	109.24	8.04
		100	86.77 ± 3.92	86.77	4.52
		300	313.74 ± 8.60	104.58	2.74
	LC-MS/MS	50	52.79 ± 1.54	105.58	2.92
		100	92.71 ± 2.87	92.71	3.10
		300	308.11 ± 5.38	102.70	1.75

Note: CV, coefficient of variation.

## Data Availability

The data that has been used is confidential.

## Supplementary Materials

The following supporting information can be downloaded at: https://www.mdpi.com/article/10.3390/bios15090625/s1. Synthesis of haptens, characterization of hapten 1–3, preparation of hybridoma cells; Figure S1. Ethical review of animal experiments; Figure S2. UV-vis absorption spectra of hapten-protein conjugates; Figure S3. Antibody subtype and purification identification; Figure S4. Optimization of AuNPs-LFIA; Figure S5. Optimized results of testing green bean samples with AuNPs-LFIA; Figure S6. Optimized results of testing leek samples with AuNPs-LFIA.; Figure S7. Parallel Analysis Results of AuNPs-LFIA and LC-MS/MS; Figure S8. Stability experiment results of AuNPs-LFIA; Table S1. Analytical performance of the three serums under homologous and heterologous source encapsulation strategies; Table S2. Detection results of 20 blind samples by AuNPs-LFIA and LC-MS/MS methods.

## Abbreviations

The following abbreviations are used in this manuscript:

CBZ	Carbendazim
AuNPs-LFIA	Gold Nanoparticle-based Lateral Flow Immunoassay
MRLs	Maximum Residue Limits
HPLC	High-performance Liquid Chromatography
LC-MS/MS	Liquid Chromatography-tandem Mass Spectrometry
LFIA	Lateral Flow Immunochromatography
DMF	N, N-dimethylformamide
CDI	N, N′-Carbonyldiimidazole
BSA	Bovine serum albumin
EDC	N-(3(dimethylamino) propyl)-N′-ethylcarbodiimide
NHS	N-hydroxysuccinimide
LF	Lactoferrin
PVP	Poly (N-vinylpyrrolidone)
NC	Nitrocellulose
UV-Vis	Ultraviolet-visible
ic-ELISA	Indirect Competitive Enzyme-Linked Immunosorbent Assay
mAb	Monoclonal antibody
SDS-PAGE	Sodium Dodecyl Sulfate Polyacrylamide Gel Electrophoresis
PB	Phosphate buffer
BB	Borate buffer
PVC	Polyvinyl chloride
T-line	Test line
C-line	Control line
LOD	Limits of detection
SD	Standard deviation
IC_50_	The half-inhibitory concentration
CR	Cross-reactivity rate
CV	Coefficient of variation
PSA	Polar surface area
MPI	Molecular Polarity Index
NMR	Nuclear Magnetic Resonance
TEM	Transmission Electron Microscope

## References

[1] Suresh I., Selvaraj S., Nesakumar N., Rayappan J.B.B., Kulandaiswamy A.J. (2021). Nanomaterials Based Non-Enzymatic Electrochemical and Optical Sensors for the Detection of Carbendazim: A Review. Trends Environ. Anal. Chem..

[2] Munir S., Azeem A., Sikandar Zaman M., Zia Ul Haq M. (2024). From Field to Table: Ensuring Food Safety by Reducing Pesticide Residues in Food. Sci. Total Environ..

[3] Xu X., Chen J., Li B., Tang L. (2018). Carbendazim Residues in Vegetables in China between 2014 and 2016 and a Chronic Carbendazim Exposure Risk Assessment. Food Control..

[4] Wang T., Wang Z., Liao G., Li X., Gu J., Qiu J., Qian Y. (2025). Carbendazim Led to Neurological Abnormalities by Interfering Metabolic Profiles in Zebrafish Brain after Short-Term Exposure. Environ. Chem. Ecotoxicol..

[5] Singh S., Singh N., Kumar V., Datta S., Wani A.B., Singh D., Singh K., Singh J. (2016). Toxicity, Monitoring and Biodegradation of the Fungicide Carbendazim. Env. Chem. Lett..

[6] (2024). Carbendazim Residue in Plant-Based Foods in China: Consecutive Surveys from 2011 to 2020. Environ. Sci. Ecotechnol..

[7] (2021). National Food Safety Standard-Maximum Residue Limits for Pesticides in Food.

[8] Wu Q., Li Y., Wang C., Liu Z., Zang X., Zhou X., Wang Z. (2009). Dispersive Liquid–Liquid Microextraction Combined with High Performance Liquid Chromatography–Fluorescence Detection for the Determination of Carbendazim and Thiabendazole in Environmental Samples. Anal. Chim. Acta.

[9] Pallavi M.S., Harischandra Naik R., Ratnamma, Nidoni U., Bheemanna M., Pramesh D. (2021). Simultaneous Determination, Dissipation and Decontamination of Fungicides Applied on Cabbage Using LC-MS/MS. Food Chem..

[10] Khosropour H., Keramat M., Laiwattanapaisal W. (2023). A dual action electrochemical molecularly imprinted aptasensor for ultra-trace detection of carbendazim. Biosens. Bioelectron..

[11] Wei X., Song W., Fan Y., Sun Y., Li Z., Chen S., Shi J., Zhang D., Zou X., Xu X. (2023). A SERS aptasensor based on a flexible substrate for interference-free detection of carbendazim in apple. Food Chem..

[12] Deng H., Cai X., Ji Y., Yan D., Yang F., Liu S., Deji Z., Wang Y., Bian Z., Tang G. (2022). Development of a Lateral Flow Immunoassay for Rapid Quantitation of Carbendazim in Agricultural Products. Microchem. J..

[13] O’Farrell B., Wong R., Tse H. (2009). Evolution in Lateral Flow-Based Immunoassay Systems. Lateral Flow Immunoassay.

[14] İnce B., Uludağ İ., Demirbakan B., Özyurt C., Özcan B., Sezgintürk M.K. (2023). Lateral flow assays for food analyses: Food contaminants, allergens, toxins, and beyond. TrAC Trends Anal. Chem..

[15] Qin J., Lu Q., Wang C., Luo J., Yang M. (2021). Colloidal gold-based lateral flow immunoassay with inline cleanup for rapid on-site screening of carbendazim in functional foods. Anal. Bioanal. Chem..

[16] Frontiers|Antibody Engineering for Pursuing a Healthier Future. https://www.frontiersin.org/journals/microbiology/articles/10.3389/fmicb.2017.00495/full?source=post_page.

[17] Zhang X., Bai Y., Tang Q., Liu M., Nan L., Wen K., Yu X., Yu W., Shen J., Wang Z. (2022). Development of epitopephore-based rational hapten design strategy: A combination of theoretical evidence and experimental validation. J. Hazard. Mater..

[18] Xie H., Li Y., Wang J., Lei Y., Koidis A., Li X., Shen X., Xu Z., Lei H. (2022). Broad-Specific Immunochromatography for Simultaneous Detection of Various Sulfonylureas in Adulterated Multi-Herbal Tea. Food Chem..

[19] Cui Y., Zhao J., Zhou J., Tan G., Zhao Q., Zhang Y., Wang B., Jiao B. (2019). Development of a Sensitive Monoclonal Antibody-Based Indirect Competitive Enzyme-Linked Immunosorbent Assay for Analysing Nobiletin in Citrus and Herb Samples. Food Chem..

[20] He F., Guo L., Liu L., Xu X., Xu C., Kuang H., Sun M. (2025). Computational Chemistry-Based Hapten Design and Antibody Production for the Immunochromatographic Assay of Maleic Hydrazide in Food and Environmental Samples. Food Chem..

[21] Dong S., Shi Q., Guan L., Wang Y., Liu P., Zhang C., Feng J. (2024). Preparation of Monoclonal Antibody and Establishment of Indirect Competitive Chemiluminescence Enzyme Immunoassay for Sodium Pentachlorophenolate. Microchem. J..

[22] Iqbal M., Usanase G., Oulmi K., Aberkane F., Bendaikha T., Fessi H., Zine N., Agusti G., Errachid E.-S., Elaissari A. (2016). Preparation of gold nanoparticles and determination of their particles size via different methods. Mater. Res. Bull..

[23] Omidfar K., Khorsand F., Darziani Azizi M. (2013). New Analytical Applications of Gold Nanoparticles as Label in Antibody Based Sensors. Biosens. Bioelectron..

[24] Liu C., Jia Q., Yang C., Qiao R., Jing L., Wang L., Xu C., Gao M. (2011). Lateral Flow Immunochromatographic Assay for Sensitive Pesticide Detection by Using Fe_3_O_4_ Nanoparticle Aggregates as Color Reagents. Anal. Chem..

[25] Raeisossadati M.J., Danesh N.M., Borna F., Gholamzad M., Ramezani M., Abnous K., Taghdisi S.M. (2016). Lateral flow based immunobiosensors for detection of food contaminants. Biosens. Bioelectron..

[26] Tan G., Zhao Y., Wang M., Chen X., Wang B., Li Q.X. (2020). Ultrasensitive Quantitation of Imidacloprid in Vegetables by Colloidal Gold and Time-Resolved Fluorescent Nanobead Traced Lateral Flow Immunoassays. Food Chem..

[27] Duan H., Chen X., Wu Y., Leng Y., Huang X., Xiong Y. (2021). Integrated Nanoparticle Size with Membrane Porosity for Improved Analytical Performance in Sandwich Immunochromatographic Assay. Anal. Chim. Acta.

[28] Tian Y., Yin X., Li J., Dou L., Wang S., Jia C., Li Y., Chen Y., Yan S., Wang J. (2024). A Dual-Mode Lateral Flow Immunoassay by Ultrahigh Signal-to Background Ratio SERS Probes for Nitrofurazone Metabolites Ultrasensitive Detection. Food Chem..

[29] Yang H., Wen H., Si Y., Ding M., Liu Y., Yu Z., Zhang L., Wang J., Pan X., Han S. (2025). Computer-Aided Precise Hapten Design Strategy for the Monospecific Detection of Altrenogest: Experimental Validation and Analysis of the Molecular Recognition Mechanism. Food Chem..

[30] Li P., Bai Y., Jiang H., Zhang Y., Li Y., Duan C., Wen K., Yu X., Wang Z. (2023). Broad-Specificity Antibody Profiled by Hapten Prediction and Its Application in Immunoassay for Fipronil and Major Metabolites. J. Hazard. Mater..

[31] Hansch C., Fujita T. (1964). P-σ-π Analysis. A Method for the Correlation of Biological Activity and Chemical Structure. J. Am. Chem. Soc..

[32] Wallace M.B., Iverson B.L. (1996). The Influence of Hapten Size and Hydrophobicity on the Catalytic Activity of Elicited Polyclonal Antibodies. J. Am. Chem. Soc..

[33] Cai X., Whitfield T., Moreno A.Y., Grant Y., Hixon M.S., Koob G.F., Janda K.D. (2013). Probing the Effects of Hapten Stability on Cocaine Vaccine Immunogenicity. Mol. Pharm..

[34] Wu W., Liu J., Mari G.M., Zhu J., Ma L., Jia L., Ding Y., Yu X., Pan Y., Wen K. (2025). Highly Sensitive ELISA for Determination of Nifursol Metabolite in Food Samples without Derivatization: From Rational Hapten Design to Molecular Recognition Mechanism. Food Chem..

[35] Ertl P., Rohde B., Selzer P. (2000). Fast Calculation of Molecular Polar Surface Area as a Sum of Fragment-Based Contributions and Its Application to the Prediction of Drug Transport Properties. J. Med. Chem..

[36] Tao Y., Zhang H., Wang Y. (2023). Revealing and predicting the relationship between the molecular structure and antioxidant activity of flavonoids. LWT.

[37] Luo L., Zhang Y., Zhao X., Wu W., Fei J., Yu X., Wen K., Shen J., Pan Y., Wang Z. (2025). Rational Hapten Design, Antibody Preparation, and Immunoassay Development for Rapid Screening Xylazine in Biological Samples. Food Chem..

[38] March D., Bianco V., Franzese G. (2021). Protein Unfolding and Aggregation near a Hydrophobic Interface. Polymers.

[39] Sinha N., Mohan S., Lipschultz C.A., Smith-Gill S.J. (2002). Differences in Electrostatic Properties at Antibody–Antigen Binding Sites: Implications for Specificity and Cross-Reactivity. Biophys. J..

[40] Pecht I., Lancet D., Pecht I., Rigler R. (1977). Kinetics of Antibody-Hapten Interactions. Chemical Relaxation in Molecular Biology.

[41] He F., Liu Y., Dai S., Sun M., Liu L., Xu L., Kuang H., Xu C., Xu X. (2025). Lateral Flow Immunochromatographic Assay Based on Computational Chemistry-Assisted Hapten Screening for the Detection of Ethoxyquin in Food Samples. Food Chem..

[42] Chen C., Huang B., Xu W., Hou R., Dong B., Yu X., Wang Z., Li H. (2024). Enhancing the Stability and Sensitivity of a Lateral Flow Immunoassay Using Stabilized AuNPs for the Simultaneous Detection of Sulfonamides and Antibacterial Synergists in Chicken Meat. Sens. Actuators B Chem..

[43] He F., Yang J., Zou T., Xu Z., Tian Y., Sun W., Wang H., Sun Y., Lei H., Chen Z. (2021). A gold nanoparticle-based immunochromatographic assay for simultaneous detection of multiplex sildenafil adulterants in health food by only one antibody. Anal. Chim. Acta.

[44] Yang D., Ma J., Xue C., Wang L., Wang X. (2018). One-pot synthesis of poly (acrylic acid)-stabilized Fe3O4 nanocrystal clusters for the simultaneously qualitative and quantitative detection of biomarkers in lateral flow immunoassay. J. Pharm. Biomed. Anal..

[45] Chen X., Lin M., Sun L., Xu T., Lai K., Huang M., Lin H. (2019). Detection and Quantification of Carbendazim in Oolong Tea by Surface-Enhanced Raman Spectroscopy and Gold Nanoparticle Substrates. Food Chem..

[46] Martins T.S., Machado S.A.S., Oliveira O.N., Bott-Neto J.L. (2023). Optimized Paper-Based Electrochemical Sensors Treated in Acidic Media to Detect Carbendazim on the Skin of Apple and Cabbage. Food Chem..

[47] Li Z., Cui Q., Li Q., Luo C., Chen M., Feng B., Li H., Bu T., Mao Y., Dang M. (2024). Preparation of Ultra-Sensitive Anti-Carbendazim Antibodies Based on New Haptens and Their Application in Lateral Flow Immunoassay. Sens. Actuators B Chem..

[48] Lan M., Guo Y., Zhao Y., Liu Y., Gui W., Zhu G. (2016). Multi-residue detection of pesticides using a sensitive immunochip assay based on nanogold enhancement. Anal. Chim. Acta.

[49] Chen Z., Tang Y., Guo P., Zhang W., Peng J., Xiong Y., Ma B., Lai W. (2024). Integration of a biocompatible metal-phenolic network and fluorescence microspheres as labels for sensitive and stable detection of carbendazim with a lateral flow immunoassay. Food Chem..

