# Supplementary material for: Haptens Optimization Using Molecular Modeling and Paper-Based Immunosensor for On-Site Detection of Carbendazim in Vegetable Products
Source: Biosensors (Basel). 2025 Sep 19;15(9):625. doi: 10.3390/bios15090625 (PMC12467735; doi:10.3390/bios15090625)
Supplement: Supplementary file 1 [file biosensors-15-00625-s001.zip › biosensors-3815040-supplementary.pdf]

biosensors

MDPI

Supplementary Material

Haptens Optimization using Molecular Modeling and Paper-Based Immunosensor for On-Site Detection of Carbendazim in Vegetable Products

Wenjing Chen [1,2], Zhuzeyang Yuan [1,2], Kangliang Pan [1,2], Yu Wang [3], Xiaoqin Yu [4], Tian Guan [1,2], Jiahong Chen [1,2,*], Hongtao Lei [1,2,*]

[1] Guangdong Provincial Key Laboratory of Food Quality and Safety / Nation-Local Joint Engineering Research Center for Machining and Safety of Livestock and Poultry Products, South China Agricultural University, Guangzhou 510642, China; wenjingchen2000@163.com (W.C.); gordon_william@163.com (Z.Y.); pkkliang@163.com (K.P.); guantian@scau.edu.cn (T.G.)
[2] Guangdong Laboratory for Lingnan Modern Agriculture, Guangzhou 510642, China
[3] Guangzhou Institute of Food Inspection, Guangzhou 511410, China; xxwangyu@163.com
[4] Sichuan Institute of Food Inspection, Key Laboratory of Baijiu Supervising Technology for State Market Regulation, Sichuan 610000, China; yuxiaoqinhuan@163.com
* Correspondence: jiahongchen@scau.edu.cn (J.C.); hongtao@scau.edu.cn (H.L.)

1. Synthesis of haptens

1.1. Hapten 1

Perform base hydrolysis of carbendazim (1 : 5 w/v in methanol) using 1 M NaOH (1 : 1 v/v) at 25°C, with TLC verification. When the product's ester group was fully hydrolyzed, the reaction was terminated. EtOAc (1 : 1 v/v) was used to extract the residue from the brine-saturated aqueous solution after the solvent was removed under vacuum and the pH was adjusted to 5.5. The final product, hapten 1, was obtained by spinning off the organic solvent after the organic phase had been sufficiently shaken and dried over anhydrous sodium sulfate.

1.2. Hapten 2

A suspension of monomethyl terephthalate (1.0 g) and CDI (0.9 g) in dry DMF (10 mL) was stirred at 25 °C for 30 min. 2-Aminobenzimidazole (0.740 g, 5.00 mmol) was introduced to the

reaction system and stirred for 12 h at 25 °C. The separation and purification were carried out using silica gel column chromatography, and the resulting product was collected by evaporation. Hapten 2 was obtained by hydrolyzing the first product using the previously described technique.

1.3. Hapten 3

A total of 1 g of butanedioic acid and 1.51 g of N, N′-carbonyldiimidazole were accurately weighed. They were dissolved in 10 mL of pyridine, and 2.33 g of potassium carbonate was added to enhance the alkalinity of the reaction system. The process was continued at room temperature for three hours. The above reaction system was spin-dried and extracted by adding primary water and dichloromethane to obtain the organic phase. The reaction process was monitored by TLC using a solvent system of dichloromethane/methanol/formic acid (v/v/v, 100:4:1), and the separation and purification were performed by silica gel column chromatography. Similarly, the hydrolysis reaction was carried out in concert with the above operation to obtain hapten 3.

2. Characterization of hapten 1-3

HPLC-MS/MS and NMR characterized hapten 1-3. For hapten 1, MS calculated for $C_8H_7N_3O_2$, 177.05, found 178.05 [M+H]+, ^{1}H NMR (600 MHz, methanol-d_4) 7.32 (dd, *J* = 5.8, 3.3 Hz, 2H), 7.18 (dd, *J* = 5.8, 3.2 Hz, 2H). For hapten 2, MS calculated for $C_{15}H_{11}N_3O_3$, 281.08, found 282.08 [M+H]+, 1H NMR (600 MHz, DMSO-d6) δ 12.67 (s, 2H), 8.26–8.19 (m, 2H), 8.08–8.02 (m, 2H), 7.46 (dd, J = 5.9, 3.2 Hz, 2H), 7.20–7.14 (m, 2H). For hapten 3, MS calculated for $C_{11}H_{11}N_3O_3$, 233.08, found 234.08 [M+H]+, 1H NMR (600 MHz, DMSO-d6) δ 7.42 (dd, J = 5.9, 3.2 Hz, 2H), 7.06 (dd, J = 5.9, 3.2 Hz, 2H), 2.68 (dd, J = 7.5, 5.6 Hz, 2H), 2.57 (dd, J = 7.4, 5.6 Hz, 2H).

3. Preparation of hybridoma cells

Three days before formal cell fusion, mice received a single injection of 100 μL of a vaccine with a dose of 1 mg/mL. First, splenocytes from immunized mice were aseptically fused with PEG 2000-induced SP2/0 myeloma cells (5:1 ratio) to perform cell fusion. Second, to remove unfused myeloma cells, the fused cells were cultivated in HAT medium for five days. On day eight, they were moved to HT medium. Third, on day 10 following fusion, the indirect competitive enzyme-linked immunosorbent assay (ic-ELISA) was used to evaluate the hybridoma supernatants' antigen binding and polymyxin inhibition. Fourth, until monoclonal colonies were verified, ELISA-positive wells were rescreened every seven days (at least three cycles) after being subcloned by limiting dilution. Ultimately, mice were given injections of the superior hybridoma cells into their peritoneal cavity to induce ascites.

4. LC-MS/MS analysis

The LC-MS/MS sample was prepared as follows: Two grams of the vegetable sample were extracted with 10 mL of a methanol/acetonitrile solution using ultrasonication for 30 min. All the solutions were filtered using a 0.22 μm filter membrane. All samples were tested within 12 h.

The liquid chromatography system was equipped with a UMISiI C18(3) column (250 × 4.6 mm) running at 40 °C with an injection volume of 10 μL and a flow rate of 0.6 mL/min. Mobile phase A is ultrapure water (with 0.1% formic acid), mobile phase B is acetonitrile. The elution conditions and mass spectrometry parameters for the three analytes were as follows:

Carbendazim (CBZ): 0–1min (A: 10%, B: 90%); 1–2.5 min (A: 20%, B:80%); 2.5–4 min (A: 30%, B: 70%); 4–6 min (A: 40%, B: 60%); 6–8 min (A: 60%, B: 40%) 8–9 min (A: 80%, B: 20%); 9–10 min (A: 90%, B: 10%). MRM transition 191.1/160.1* and 191.1/132.1 (m/z, *Quantitative ion pair), declustering potential (DP) both 68.2 V, and collision energy (CE) 17.4 and 27.3 eV, respectively.

Mass spectrometer settings were all in ion mode, positive ionization; ion source, electrospray ionization (ESI); or multiple reaction monitoring (MRM) mode.

華南農業大學
South China Agricultural University

Results of Ethical Review of Animal Experiments

No.（2024B094）

Experiment item	Research on Key Technologies for Multidimensional Identification and Detection of Food Authenticity			
Application number	2024B094			
Comments on conservation of experimental animals	All the experimental mice used in this experiment came from an experimental animal center with legal license. The type, quantity, and grouping of mice were conformed to the 3R principle.			
Comments on welfare evaluation of experimental animals	This experiment was carried out in a laboratory with a license for experimental animals which was confirmed to the welfare principle.			
Comments on ethics and morality	The animals were euthanized after the experiment.			
Comments on comprehensive scientific evaluation	This experimental study has scientific significance.			
Experimental time, animal species and quantity	Date: 2024-07-01 to 2024-09-01<br>Animal species: BALB/c mice (SPF grade)<br>Quantity: 45 females			
Comments of the ethical reviewer	Agree.			
	Reviewer	Rangcai Yu	Review Date	2024-06-26
Comments of the ethical reviewer	Agree.			
	Reviewer	Wei Huang	Review Date	2024-06-28
Final comments of the director （or deputy director）	Agree.			
	Reviewer	Zhonghua Liu	Review Date	2024-07-03

Experimental Animal Ethical Committee of South China Agricultural University

Date: [illegible]

Figure. S1 Ethical review of animal experiments.

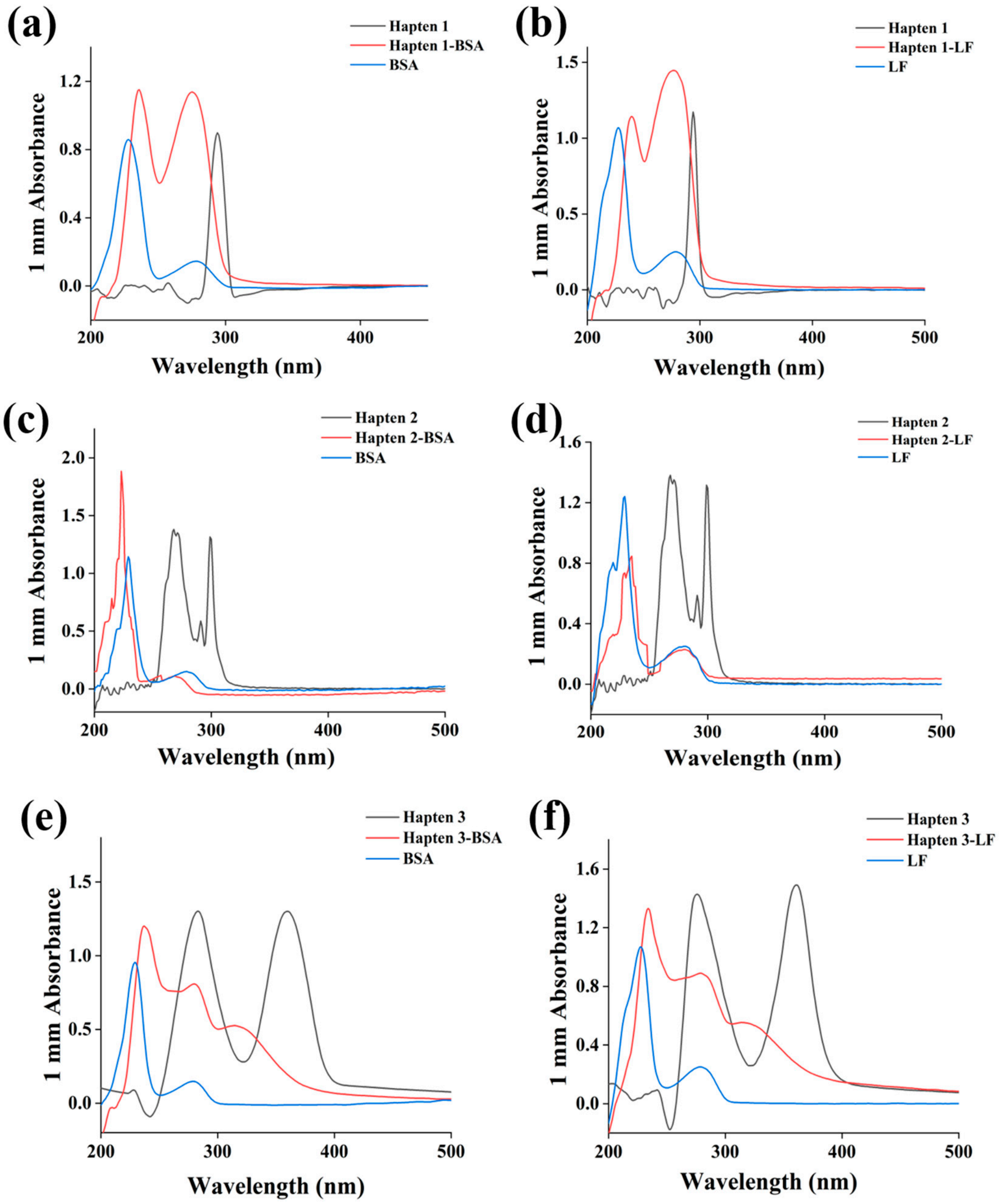

Figure. S2 UV–vis absorption spectra of (a) BSA, Hapten 1, Hapten 1-BSA; (b) LF, Hapten 1, Hapten 1-LF (c) BSA, Hapten 2, Hapten 2-BSA; (d) LF, Hapten 2, Hapten 2-LF; (e) BSA, Hapten 3, Hapten 3-BSA, and (f) LF, Hapten 3, Hapten 3-LF.

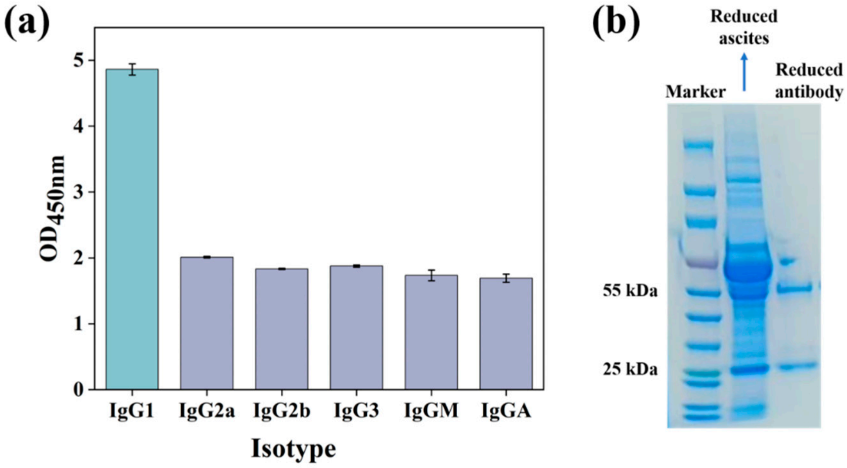

Figure. S3 (a) Determination of antibody isotypes; (b) verification of antibody purification by SDS-PAGE.

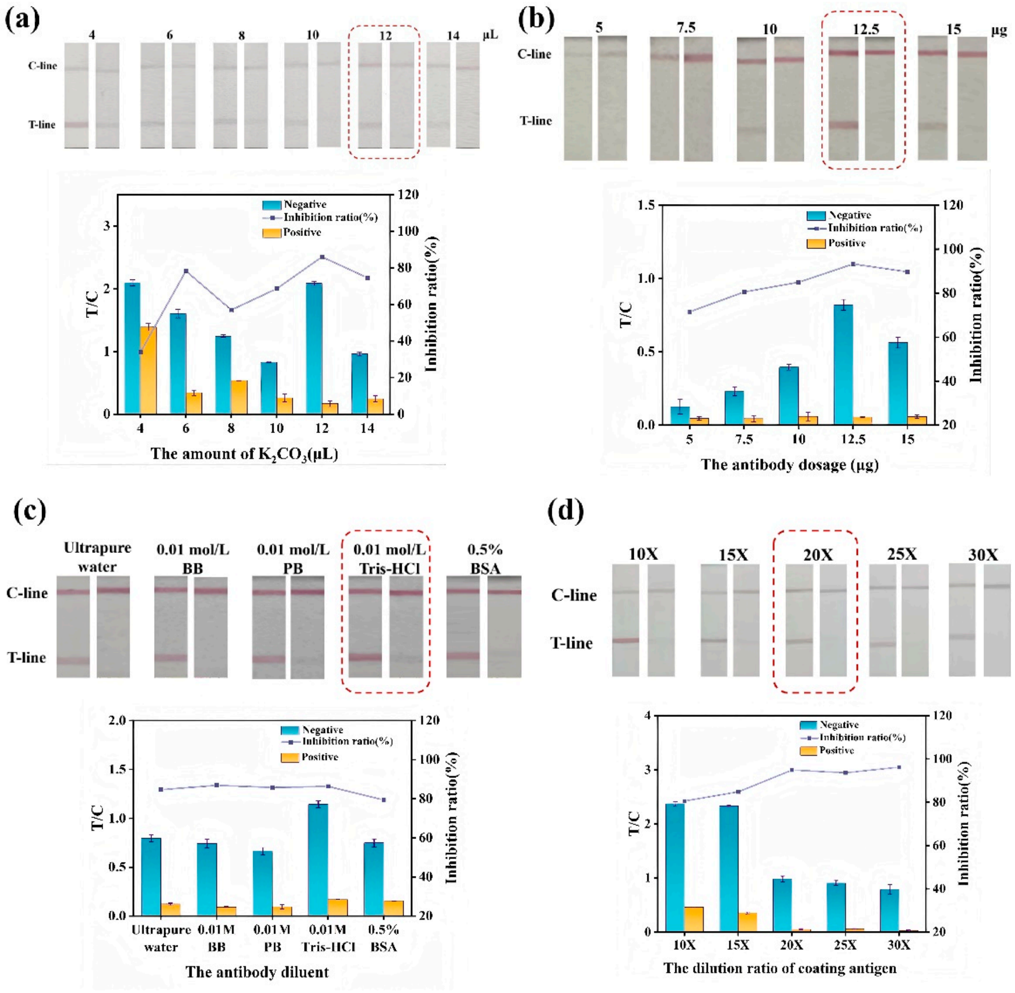

Figure. S4 Optimization of AuNPs-LFIA. (a) pH for labeling; (b) antibody amount for labeling; (c) dilution buffer of antibody; and (d) concentration of Coating Antigen.

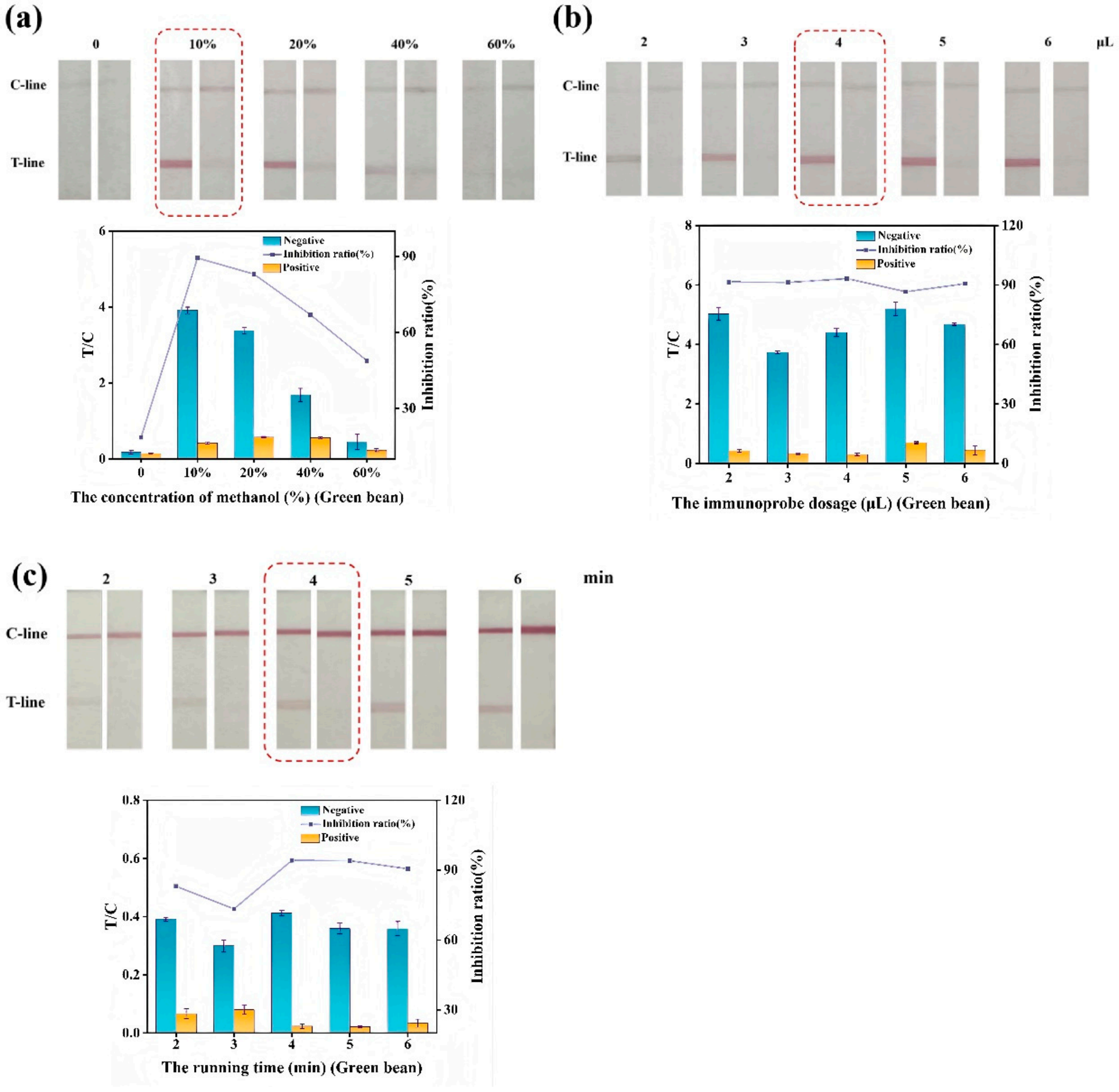

Figure. S5 Optimized results of testing green bean samples with AuNPs-LFIA. (a) The concentration of methanol, (b) the immunoprobe dosage, and (c) the running time.

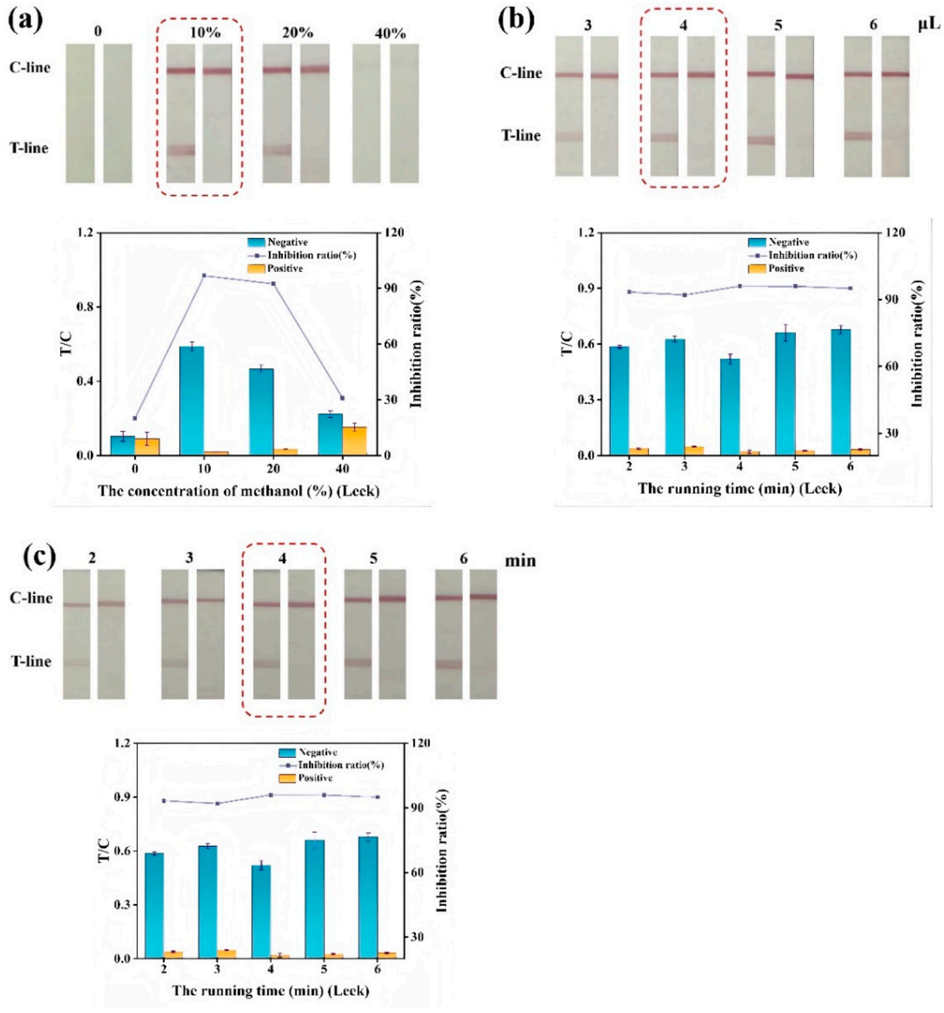

Figure. S6 Optimized results of testing leek samples with AuNPs-LFIA. (a) The concentration of methanol, (b) the immunoprobe dosage, and (c) the running time.

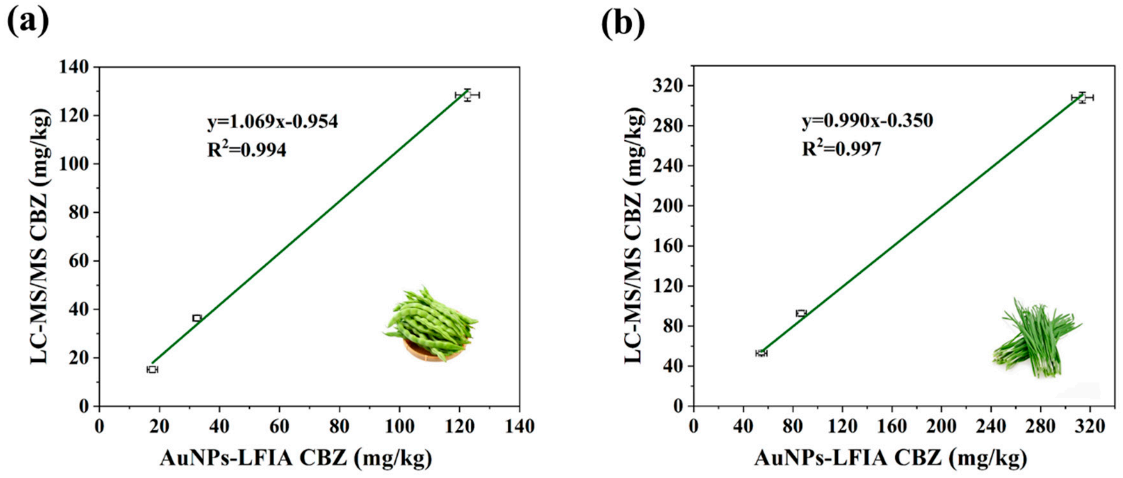

Figure. S7 Parallel analysis results of AuNPs-LFIA and LC-MS/MS. (a) Parallel analysis results for green beans, and (b) parallel analysis results for leeks.

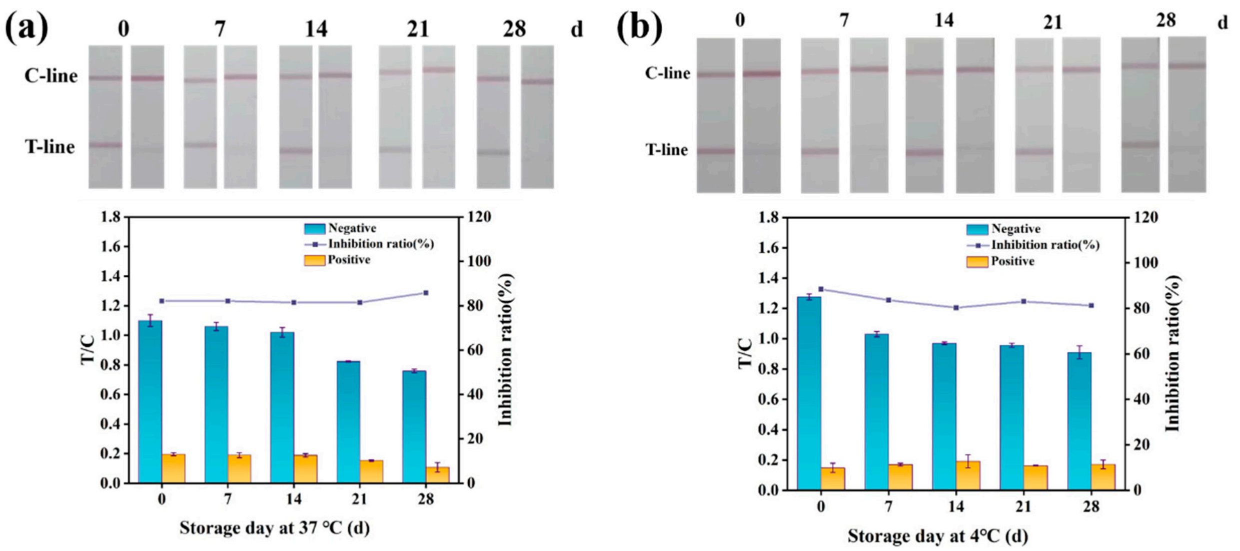

Figure. S8 Stability experiment results of AuNPs-LFIA. (a) Accelerated stability experiment; (b) low temperature stability test.

Table S1 Analytical performance of the three serums under homologous and heterologous source encapsulation strategies.

Number of Serum	Coating antigen	Titer	Inhibition rate (%)
Hapten 1-LF-1	Hapten 2-BSA	1:32000	78.46%
	Hapten 3-BSA	1:16000	76.44%
	Hapten 1-BSA	1:32000	73.88%
Hapten 1-LF-2	Hapten 2-BSA	1:16000	74.56%
	Hapten 3-BSA	1:16000	70.30%
	Hapten 1-BSA	1:8000	71.48%
Hapten 1-LF-3	Hapten 2-BSA	1:16000	75.55%
	Hapten 3-BSA	1:8000	60.02%
	Hapten 1-BSA	1:8000	67.88%
Hapten 2-LF-1	Hapten 2-BSA	1:16000	47.55%
	Hapten 3-BSA	1:8000	56.43%
	Hapten 1-BSA	1:32000	60.12%
Hapten 2-LF-2	Hapten 2-BSA	1:16000	50.94%
	Hapten 3-BSA	1:4000	62.30%
	Hapten 1-BSA	1:8000	55.31%
Hapten 2-LF-3	Hapten 2-BSA	1:16000	44.32%
	Hapten 3-BSA	1:8000	62.11%
	Hapten 1-BSA	1:8000	59.89%
Hapten 3-LF-1	Hapten 2-BSA	1:16000	32.22%
	Hapten 3-BSA	1:8000	59.15%
	Hapten 1-BSA	1:16000	22.13%
Hapten 3-LF-2	Hapten 2-BSA	1:32000	40.51%
	Hapten 3-BSA	1:16000	53.55%
	Hapten 1-BSA	1:8000	30.13%
Hapten 3-LF-3	Hapten 2-BSA	1:16000	28.75%
	Hapten 3-BSA	1:16000	45.46%
	Hapten 1-BSA	1:8000	27.82%

Note: the inhibitory drug (carbendazim) concentration was 500 ng/mL.

Table S2 Detection results of 20 blind samples by AuNPs-LFIA and LC-MS/MS methods (n=3).

Sample	Sample number	AuNPs-LFIA (μg/kg)	LC-MS/MS (μg/kg)
Green beans	1	-	ND
	2	-	ND
	3	-	ND
	4	-	ND

	5	-	ND
	6	535±4.47	567±1.24
	7	-	ND
	8	-	ND
	9	-	ND
	10	-	ND
	11	-	ND
	12	2580±6.27	2649±4.33
	13	-	ND
	14	-	ND
Leek	15	-	ND
	16	-	ND
	17	-	ND
	18	-	ND
	19	-	ND
	20	-	ND

Note: ND, negative; -, unavailable; Samples 1-10 were green beans, and samples 11-20 were leeks.
